# Namibian fairy circles: Hostile territory for soil nematodes

**DOI:** 10.1371/journal.pone.0315884

**Published:** 2025-08-12

**Authors:** Amy Treonis, Andrew Bell, Eugene Marais, Gillian Maggs-Kölling

**Affiliations:** 1 Department of Biology, University of Richmond, Richmond, Virginia, United States of America; 2 Teaching and Scholarship Hub, University of Richmond, Richmond, Virginia, United States of America; 3 Gobabeb Namib Research Institute, Windhoek, Namibia; 4 Unit for Environmental Sciences and Management, North-West University, Potchefstroom, South Africa; University of Limpopo, SOUTH AFRICA

## Abstract

Fairy circles are rings of grass (2–12 m diameter) with centers of bare soil that are found in some arid grasslands. Above- and belowground chemical and biological processes have been explored in an ongoing debate about the ultimate causes of this pattern. Belowground biota, including beneficial and pathogenic nematodes in the soil food web, may both influence and respond to the formation of fairy circles. For example, root-feeding nematodes have the potential to enhance plant water stress, promoting the spatial organization into rings to minimize competition. We studied the soil nematode communities associated with *Stipagrostis* fairy circles along a 900-km range in the Namib Desert of Namibia in southern Africa. Nematode abundance and diversity were highest in soils beneath the vegetation rings that define fairy circles and in soils in the vegetated matrix surrounding the rings, demonstrating the positive impact of plant-derived resources (i.e., roots and organic matter) on nematode communities. Network analysis of nematode communities showed that in many ring soils, fungal or root hair-feeding *Aphelenchoides*, *Ditylenchus*, and *Hexatylus* frequently co-occurred among other plant-parasitic taxa. In contrast, soils from the bare centers of fairy circles had lower organic matter content and were nearly defaunated. Fairy circle centers appear to be a hostile environment for soil nematodes, reflecting a resource-limited soil food web that may contribute to the persistent absence of vegetation. Bacterial-feeding *Acrobeloides* were over-represented in communities in the centers, which could reflect an association with termites or ants, whose activity has been proposed to play a role in fairy circle formation. These findings show that nematode communities respond to the unique environmental conditions created by fairy circles, and nematode assemblages found in center, ring, and matrix soils also may contribute to sustaining the pattern.

## Introduction

**Fig 1:**
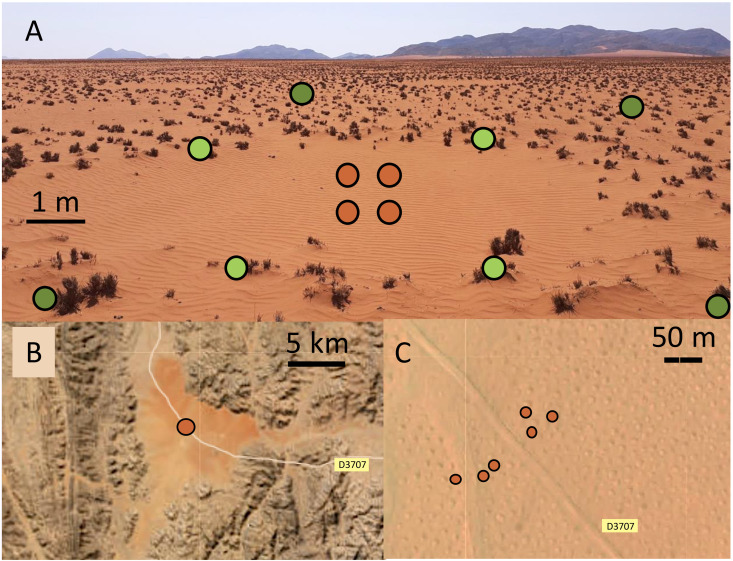
Fairy circles in the Giribes Plains, Kunene region, northwest Namibia. (A) A fairy circle. Brown dots represent sampling in the “center”, light green dots in the “ring”, and dark green dots in the surrounding “matrix”. (B) & (C) Satellite views of the Giribes Plains, Namibia, with fairy circle sampling sites marked in brown [8].

Fairy circles are a unique and enigmatic vegetation pattern found in some arid grasslands. Originally reported from Namibia [1–4], they have also been found in Australia [5] and elsewhere in the world [6]. This pattern consists of regularly spaced, bare circular patches (2–12 m diameter) that are each surrounded by a ring of grass that may have enhanced growth relative to the grasses and forbs that grow between the rings (Fig 1). Fairy circles formed by grasses in arid ecosystems are not related to the fungal induced “fairy rings” found elsewhere. In southern Africa, fairy circles form in windblown, sandy soils with 50–150 mm annual precipitation and occur along a range extending from Angola, through Namibia, to South Africa [2–4,7]. This area is the transition zone between the sparsely vegetated gravel plains and sand dunes of the Namib Desert to the west and the arid shrub savanna of the interior. The grasses associated with fairy circles generally are perennial species of *Stipagrostis* [4,7] that provide forage for wildlife, including zebra, oryx, springbok, and ostrich.

The mechanisms driving the formation of fairy circles are the subject of active debate and research [4,6,9–11]. One leading hypothesis is that *Stipagrostis* grasses self-organize into rings due to belowground competition for water and nutrients [4,11–15]. It also has been proposed that the activity of subterranean termites or ants creates the bare patches through soil modification or foraging behavior [2,9,10,16–20]. Various other processes also have been considered, including allelopathic effects from *Euphorbia* shrubs [21,22] and the impact of microbial pathogens [23,24]. Ultimately, fairy circles may arise from the interaction of multiple ecological processes [6,9,25].

To date, studies of the biological and hydrological properties of fairy circles have overlooked soil nematodes, which are abundant in desert soils [26–29]. Nematodes rely on water films for movement, feeding, and reproduction [30], but under dry conditions, they can enter an inactive and ametabolic state known as anhydrobiosis [31]. Soil nematodes in anhydrobiosis can rapidly (within minutes or hours) become active in response to rainfall [32], melting snowfall [33], and fog that wets the soil [29]. Soil organic matter is the primary factor affecting nematode abundance and diversity in desert soils, rather than a lack of moisture or extreme temperatures [28,29,34,35].

As integral members of the soil food web, nematodes contribute to decomposition and nutrient cycling in soils [36]. They rely on plant-derived resources either directly, as plant-parasites, or indirectly, as consumers of decomposer bacteria and fungi [37]. Plant-parasitic taxa negatively affect plant health by damaging root systems, and their presence may contribute to plant water stress [38]. On the other hand, the activity of microbivorous nematodes (bacterial and fungal feeders) is known to enhance nutrient availability in soils, which positively supports plant growth [39]. Soil nematode communities are very sensitive responders to changes in soil properties and vegetation patterns [28,40–42], and fairy circles alter a range of soil characteristics that are likely to affect nematodes, including organic matter, particle size, pH, salinity, moisture, and temperature [2,15,24,25,43–45]. Prior studies have shown that soil bacterial, archaeal, and fungal communities differ significantly between the bare centers and vegetated rings of fairy circles, with lower microbial biomass and diversity in the centers [23,24,46]. Soil nematodes may be similarly responsive, and their communities may provide insights into how soil habitability is affected in fairy circle landscapes.

The objective of this study was to investigate the diversity and abundance of soil nematodes associated with fairy circles. We sampled soils from fairy circles at nine sites in the Namib Desert to compare nematode communities and soil properties in the bare centers, vegetated rings, and surrounding matrix. We hypothesized that nematode communities would vary across these microhabitats due to their dependence on plant-derived resources. We predicted that nematode abundance would be highest in vegetated ring and matrix soils compared to the bare centers. We also predicted that these communities would have increased numbers of plant-parasitic and fungal-feeding taxa, due to the presence of roots and high organic matter content. In addition to quantifying nematode abundance and diversity, we used network analysis to identify co-occurring assemblages associated with positions across the fairy circles. Our findings suggest that nematode communities that are found in center, ring, and matrix soils reflect, and potentially reinforce, the ecological dynamics that sustain fairy circles.

## Materials and methods

### Study sites

**Fig 2:**
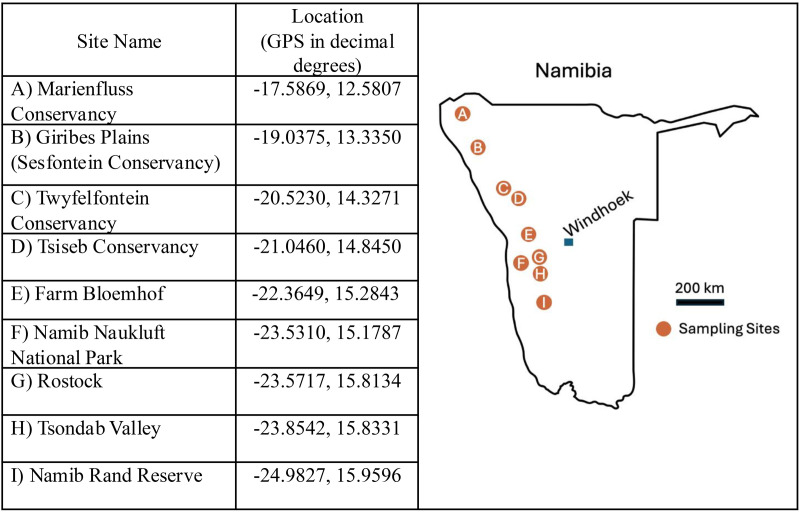
Namib Desert fairy circle sites that were sampled for study of soil nematode communities.

Nine fairy circle sites were sampled, spanning a 900-km north-south range that encompasses most of the distribution of fairy circles in Namibia [4,7] (Fig 2). Eight sites were along the eastern verge of the Namib Desert, and the remaining site (Namib Naukluft) was within the most arid core of the desert. Due to drought, there was little fresh plant growth at any of the sites, and most of the grass was grazed to short stubs (Fig 1). It was not possible to identify the *Stipagrostis* species present in the rings due to the absence of structures (e.g., leaves, inflorescences). At some sites (Namib Rand, Tsiseb), a small number of forbs and other grasses were visible in the matrix area between fairy circles at the time of sampling. During non-drought periods, plants grow in the rings and throughout the matrix, while the centers of the fairy circles remain devoid of vegetation [4] (Fig 1). Soils were sandy Eutric Regosols (Tsiseb, Farm Bloemhof, Rostock, Namib Rand) or Petric Calcisols (Marienfluss, Giribes, Twyfelfontein, Namib Naukluft, Tsondab). Soils were collected under a research permit issued to A. Treonis (RPIV01022019) by the Namibia National Commission on Research Science and Technology. Permission to work in sampled areas was provided by the Namibian Ministry of Environment, Forestry and Tourism (Namib Naukluft, Tsondab Valley), the Namib Rand Nature Reserve (Namib Rand), private landowners (Farm Bloemhof, Rostock), and the communal conservancies.

### Soil sampling

Soils were collected between 24 March – 31 October 2020. Due to travel restrictions during the Covid-19 pandemic, sampling could not be completed within a short time frame. However, due to severe drought conditions, very little rainfall occurred in the Namib Desert over the sampling period that would stimulate plant growth and nematode communities [29]. Furthermore, the study sites are outside of the area of the Namib Desert that is wetted by fog [29]. Thus, nematodes in these soils would have mostly been inactive throughout the study period (i.e., anhydrobiotic and not feeding or reproducing). A review of the use of anhydrobiosis in soils suggest that nematodes are inactive when moisture content falls below 2% [47], and the soils sampled from fairy circles for this study all were < 1% soil moisture at the time of sampling. It is also not likely that nematode community composition would have changed over the study period. Freckman and Mankau [48] showed that seasonal changes in nematode abundance in Mojave Desert soils were linked to rainfall, but they also found that the relative contribution of nematode trophic groups was unchanged over the study period.

Six representative circles were selected at each site within an approximately 1 ha area. At each circle, four subsamples were collected from the center of the circle (“center”) to a depth of 0–10 cm using a plastic scoop and combined (Fig 1). Similarly, four subsamples were collected and combined from the soil beneath the grass composing the ring of vegetation defining the fairy circle (“ring”), and four subsamples were collected and combined from the inter-circle matrix (“matrix”), at least 1 m from the ring (Fig 1). Matrix samples were collected from beneath vegetation, but the plants tended to be smaller than those defining the ring. A total of 162 samples were collected (nine sites x six rings x three positions). Soils were sieved (2-mm mesh) to remove rocks and transferred to plastic bags. The dry, sandy soil contained very few rocks and passed easily through the sieve, minimizing any physical impact on nematodes.

### Soil analyses

Soil moisture was determined gravimetrically (24 h at 105°C [49]). Soil organic matter was measured as loss on ignition (LOI, 360°C, 2 h [50]). Suspensions of 10-g air-dried soil in 30-ml deionized H_2_O were mixed and equilibrated for 30 min before solution pH was measured with a Pocket Pro + pH Tester (Hach, Loveland, CO). Using the same solution, electrical conductivity (EC) was measured as an indicator of salinity using a conductivity meter (Traceable^®^, Cole-Parmer, Vernon Hills, IL [29]). Desert soils accumulate salts due to low precipitation and high evaporation, which can impact the biological availability of moisture to soil organisms.

### Nematode community analyses

Nematodes were extracted over 72 h from 120 g fresh soil using a Baermann funnel technique (60 g soil x 2 funnels per sample, combined when drawing off the solution [51]). This extraction method relies on nematode movement but allows sufficient time for anhydrobiotic nematodes to revive. After extraction, sample volume was reduced to approximately 500 µl, and nematodes were fixed in 5% formalin solution [52]. Nematodes were counted and identified to the lowest taxonomic level possible using a Zeiss inverted microscope (Carl Zeiss, Inc., White Plains, NY). Most nematodes were identified to the genus level, although certain groups with very low abundance and that were only found as juveniles could not be confidently identified at low taxonomic levels (i.e., dorylaims and plant-parasites in the family Dolichodoridae). Bongers [53] and Andrássy [54] were consulted for assistance with nematode identification. Nematode taxa were assigned to trophic groups according to Yeates et al. [37]. Nematode abundance data were used to calculate nematode density (# 100 g^-1^ dry soil) and the Simpson’s Diversity Index [55]. Simpson’s Index is less sensitive to rare species, offering a more conservative measure of diversity when comparing communities. The proportional representation of bacterial-feeding nematodes in the microbivore community was calculated as B/(B + F), where B was the number of bacterial-feeders and F was the number of fungal-feeders in the sample. Bongers [56] was used to assign values (1–5) to the nematode taxa on the colonizer-persister (cp) scale. Low cp-values are assigned to taxa with high reproductive rates that respond rapidly to pulses of enrichment. Higher cp-values are given to taxa with low reproductive rates and longer life cycles that are likely to be more abundant in stable habitats.

### Statistical analyses

Statistical analyses were performed with R version 4.4 (https://www.r-project.org) [57]. Analysis of variance (ANOVA) was used to investigate differences in soil organic matter content and electrical conductivity (log-transformed) among sites and positions in the fairy circles. Means were compared using Tukey’s Honestly Significant Difference (HSD) multiple comparison procedure. Prior to ANOVA, the Shapiro-Wilk test was used to assess all dependent variables for normality, and Levene’s test was used to test for unequal variance. Variables that could not be transformed to a normal distribution or that exhibited heteroscedasticity (nematode abundance, soil moisture, pH, B/(B + F), Simpson’s Index) were analyzed using the Kruskal-Wallis nonparametric test, followed by Dunn’s test for multiple comparisons, with P-values adjusted with a Bonferroni correction. Spearman’s correlation coefficient was calculated to investigate relationships among variables.

To evaluate how much of the variation in nematode community composition could be explained by site, fairy circle position, and standardized soil variables, we conducted a constrained linear ordination using redundancy analysis (RDA; [58]) in the R *vegan* package [58]. Species data were Hellinger-transformed prior to analysis to reduce the influence of rare taxa and meet the assumptions of linear ordination. The RDA model was constructed using forward selection of variables, which excluded soil moisture and organic matter in favor of those that explained a significant proportion of the variation (site, position, electrical conductivity, and pH). Five samples containing no nematodes were omitted from the analysis (one each from Rostock, Tsondab, and Farm Bloemhof, and two from Tsiseb; all from the center position). To statistically assess differences in nematode community composition among sites and positions, we also performed a permutational multivariate analysis of variance (PERMANOVA) using a Bray-Curtis dissimilarity matrix, based on 999 permutations, implemented with the *adonis2* function in the *vegan* package [59].

To identify assemblages of co-occurring nematode taxa at each of the fairy circle positions, we used a co-occurrence network analysis approach. We excluded taxa represented by ten or fewer nematodes in the dataset (a natural cut-off point) that may be transient members of the community. Thresholds of 0.3 (positive correlations) and −0.2 (negative correlations) were applied to filter for the most statistically meaningful correlations (P < 0.05) from the Spearman’s correlation matrix of taxa abundances, resulting in an adjacency matrix that served as the basis for our network graph (S3 Table). An undirected graph was built for each position using the Python NetworkX package, where nodes represent the nematode taxa and edges (e.g., lines) indicate significant correlation between the abundances of two taxa (positive or negative). Several centrality measures were computed to quantify the importance of each taxon within the network. Degree centrality reflects the number of direct connections (i.e., significant correlations) each taxon has and was used to determine the node size for each taxon in the network. Betweenness centrality reflects the degree to which specific taxa bridge between other taxa in the network and is reflected in the thickness of the edges between nodes. Closeness centrality is indicated by shared node color and identifies taxa/nodes that are highly connected to each other, indicating that they co-occur frequently. Community detection was performed using the Girvan-Newman algorithm [60] to identify clusters of species that interact more frequently with each other than with those outside their community.

## Results

### Soil properties

Across the study sites, all soils were very dry with low organic matter content (Table 1, S1 Table). Considering soils from all the sites collectively, soil from the ring and matrix contained more organic matter than soil from the centers of the fairy circles (ANOVA, F_2,159_ = 16.09, P < 0.001, Table 1). However, there were some sites where the differences were not significant between the center and the ring (Namib Naukluft, Rostock, and Namib Rand) or the center and the matrix (Tsiseb, FarmBloemhof, Rostock, and Tsondab) (S1 Table). Moisture did not differ among fairy circle positions (Kruskal–Wallis test: χ² = 0.98, df = 2, P = 0.61, Table 1, S1 Table), except at Tsiseb where center soils were higher than those from the ring (S1 Table). Electrical conductivity (EC) was lowest in the center soils, followed by the matrix, and highest in the ring (ANOVA, F_2,159_ = 55.03, P < 0.001, Table 1). Within two sites, these differences were not significant (Rostock and Tsondab, S1 Table). pH did not differ among fairy circle positions (Kruskal–Wallis test: χ² = 3.68, df = 2, P = 0.16, Table 1, S1 Table), except at Giribes where the center was significantly more basic than the ring or matrix (S1 Table).

**Table 1:** Soil properties in fairy circle soils.

Position in Fairy Circle	Organic matter content (%)^*^	Soil moisture (g 100 g^-1^)	Electrical Conductivity (EC) µS cm^-1^^*^	pH
Center	0.26 ± 0.02a	0.33 ± 0.02a	100.8 ± 5.3a	8.3 ± 0.08a
Ring	0.37 ± 0.02b	0.32 ± 0.02a	171.9 ± 12.1c	8.1 ± 0.08a
Matrix	0.34 ± 0.01b	0.31 ± 0.02a	134.9 ± 6.6b	8.2 ± 0.07a

* Indicates significant differences among fairy circle positions (ANOVA or Kruskal Wallis, n = 54 samples per position). Lowercase letters indicate differences among the positions (Tukey’s HSD or Dunn’s Test). Soil properties for each site can be found in S1 Table.

### Nematode abundance

**Fig 3:**
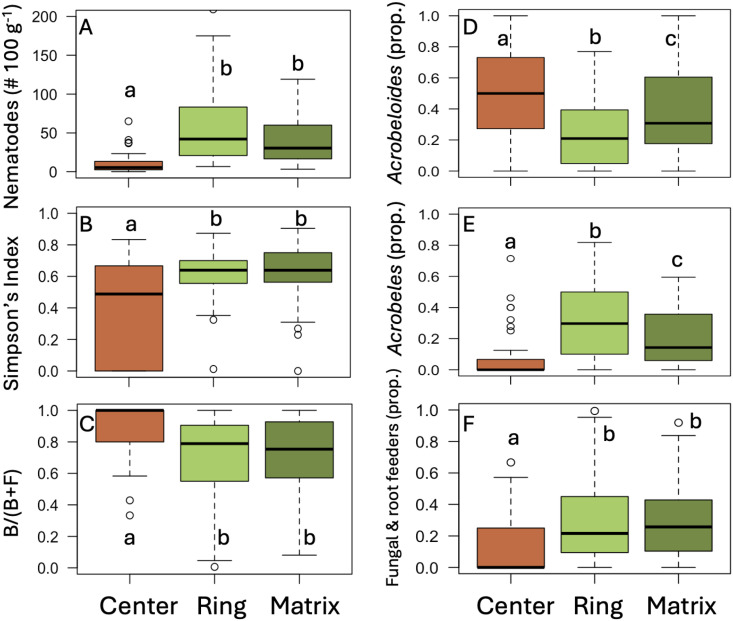
Nematode abundance and diversity in fairy circle soils collected from the center of circles, from the grass ring, or from the surrounding matrix. (A) Total nematode abundance. (B) Simpson’s Diversity Index. (C) Bacterial-feeders [B/(B + F)]. (D) *Acrobeloides* (the most common nematode across the sites). (E) *Acrobeles* (the second most common). (F) Fungal and root feeding taxa. Boxplots represent the interquartile range with lines representing the median value. Whiskers represent the minimum/maximum values, and circles are outliers. n = 54 samples per position, collected from nine field sites. Lowercase letters indicate statistically significant differences among positions (ANOVA, Tukey’s HSD Test or Kruskal-Wallis and Dunn’s Test, P < 0.001).

Total nematode abundance ranged from 0–309.2 100 g^-1^ soil (n = 162). Considering soils from all sites collectively, center soils contained significantly fewer nematodes than ring or matrix soils (Kruskal–Wallis test: χ² = 71.38, df = 2, P < 0.001, Fig 3A, S1 Fig). There were some sites where the differences in nematode abundance were not significant between the center and the ring (Twyfelfontein, Tsiseb, FarmBloemhof, and Namib Naukluft,) or the center and the matrix (Marienfluss, FarmBloemhof and Tsondab, and Namib Rand) (S1 Fig). Nematode abundance was correlated positively to organic matter content (Spearman’s correlation, ρ = 0.35, P < 0.001) and EC (ρ = 0.62, P = 0.039) and negatively to pH (ρ = −0.17, P = 0.026). Abundance was not correlated to moisture content (ρ = −0.12, P = 0.12).

### Nematode diversity

Nineteen taxa of nematodes were identified across the sites (S2 Table). *Acrobeles*, *Acrobeloides*, *Aphelenchus*, and *Aphelenchoides* were the only taxa present at all nine sites (S2 Table). Several taxa were rare, with fewer than ten individuals seen across the entire study (*Carcharolaimus, Discolaimus, Drilocephalobus, Nothacrobeles, Pelodera,* and *Plectus*). Richness of nematode communities ranged from 0–10 taxa. Considering soils from all the sites together, richness and the Simpson’s Diversity Index were both higher in ring and matrix soils than in soils from the fairy circle centers (Kruskal–Wallis test: χ² = 65.97, df = 2, P < 0.001 for richness, χ² = 13.49, df = 2, P = 0.001 for Simpson’s, Fig 3B). These differences were not significant when considering many of the sites individually (S2 Fig). Richness was positively correlated to organic matter content (Spearman’s correlation, ρ = 0.30, P < 0.001) and EC (ρ = 0.17, P = 0.028), which were higher in ring and matrix soils (Table 1). The Simpson’s Index was not correlated to organic matter (ρ = 0.08, P = 0.29) or EC (ρ = 0.07, P = 0.35). Instead, the Index was negatively correlated to both moisture (ρ = −0.20, P < 0.011) and pH (ρ = −0.28, P = 0.0003), factors that did not vary significantly among the positions. Nearly all the nematodes found were assigned cp-values of 1 or 2 (S2 Table).

### Nematode community structure

Nematode communities in most soil samples were dominated by bacterial-feeding taxa, with the proportion of the microbivore community composed of bacterial-feeders [B/(B + F)] significantly higher in the center soils than in ring or matrix (Kruskal-Wallis Test, χ² = 29.94, df = 2, P < 0.001, Fig 3C). *Acrobeloides* and *Acrobeles* were the most common nematodes found (i.e., 49% of nematodes identified). Their representation differed among fairy circle positions, with *Acrobeloides* over-represented in center compared to ring and matrix soils (Kruskal-Wallis Test, χ² = 15.70, df = 2, P = 0.0039, Fig 3D) and *Acrobeles* under-represented (Kruskal-Wallis Test, χ² = 49.77, df = 2, P < 0.001, Fig 3E). The proportion of the nematode community consisting of fungal-feeders, root-feeders, and plant-parasites (S2 Table) was higher in ring and matrix soils than in center soils (Kruskal-Wallis Test, χ² = 28.41, df = 2, P < 0.001, Fig 3F).

**Fig 4:**
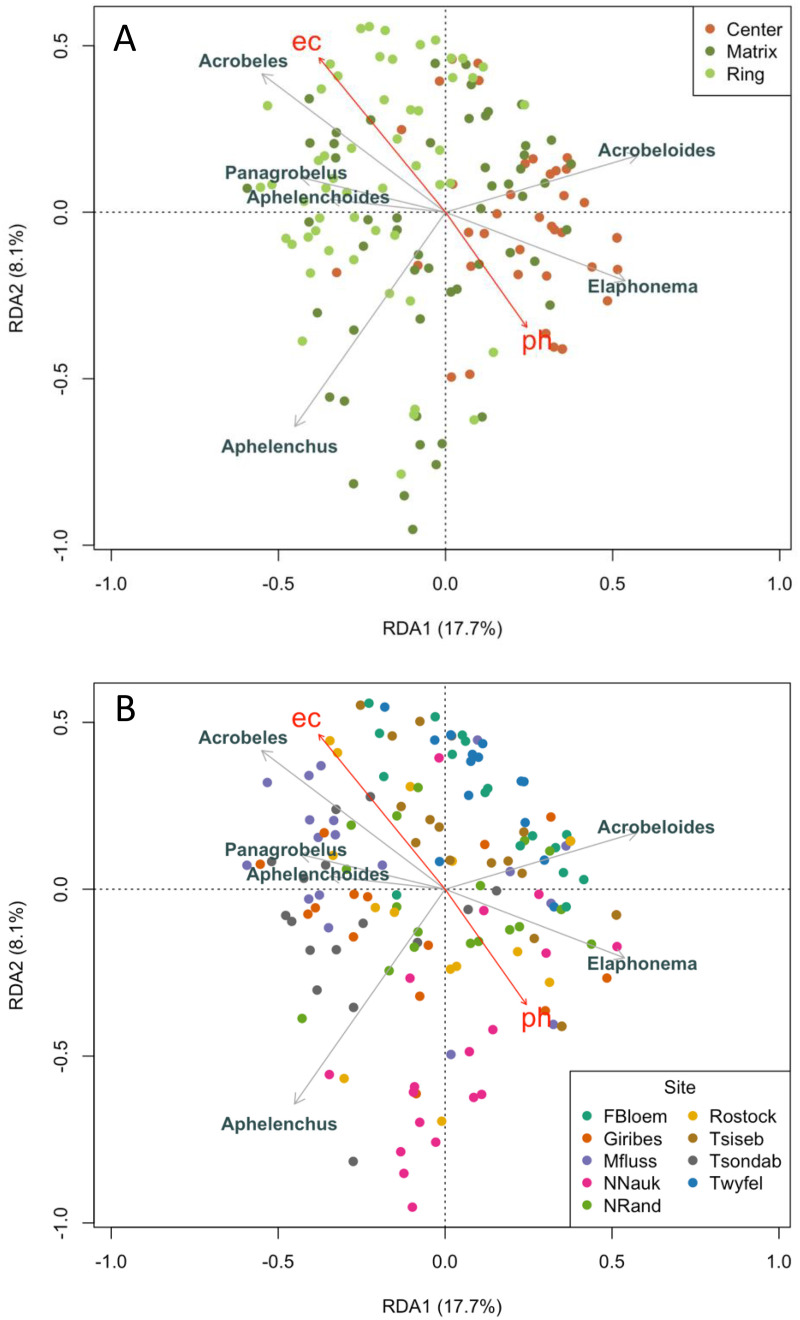
Ordination biplots for RDA of relationships among soil properties and nematode taxa in fairy circle soils. Circles represent individual soil samples (n = 157 soils that contained nematodes). Red vectors represent the effects of environmental variables that explained significant amounts of variation [pH, and EC (electrical conductivity)]. Nematode taxa and their vectors are in gray. Only the six taxa with the longest vectors are labeled to reduce visual clutter and highlight those most strongly associated with the primary RDA axes. The constrained axes explained a significant proportion of the variation in nematode communities. A) Samples are color-coded to reflect their position in the fairy circles. B) Samples are color-colored by site.

RDA elucidated the contribution of environmental factors to differences in community composition among positions and sites (Fig 4). The first canonical axis (RDA1) explained 43.2% of the constrained variance (approximately 17.7% of the total variance), while the second axis (RDA2) explained an additional 19.7% of the constrained variance (8.1% of total). Together, RDA1 and RDA2 accounted for 62.9% of the explained variation, capturing the primary environmental gradients influencing community structure. The full RDA model explained 40.9% of the total variance in species composition, indicating that the environmental gradients represented by site, position, pH, and EC are important drivers of nematode community composition across the study sites. The remaining 59.1% of the variation was unconstrained and likely reflects residual variation or unmeasured factors. Organic matter and soil moisture were tested but did not significantly explain variation and were excluded from the final model. Among included predictors, site was the strongest (36.55% of constrained variation; variance = 0.108, F_8,144_ = 7.23, P = 0.001), followed by position (23.14%; variance = 0.068, F_2,144 _= 18.32, P = 0.001), with smaller contributions from pH (1.56%; variance = 0.005, F_1,144 _= 2.47, P = 0.018) and EC (1.76%; variance = 0.005, F_1,144 _= 2.77, P = 0.011).

Nematode community structure was significantly influenced by both site and fairy circle position, with a strong interaction between the two (PERMANOVA, site x position F_16,156 _= 3.622, P = 0.001). Center soils had different nematode communities from the ring and matrix positions, with Farm Bloemhof as the only site lacking this separation (Fig 4). Site accounted for slightly more of the variation in community composition than position (24.2% vs. 18.5%, PERMANOVA). RDA showed that the bacterial-feeding genera *Panagrobelus* and *Acrobeles* and the fungal-feeder *Aphelenchoides* were associated with soils characterized by higher electrical conductivity and lower pH, which were mainly matrix and ring soils (Fig 4A). In contrast, *Elaphonema* was associated with higher pH soils and the center position, and *Acrobeloides* was also more abundant in centers (Fig 4A). At the site level, *Aphelenchus* (a fungal-feeder) was more common at Namib-Naukluft, while *Acrobeloides* was more abundant at Twyfelfontein and Farm Bloemhof (Fig 4B). RDA did not detect strong environmental associations for the remaining taxa, which were comparatively rare. A substantial proportion of the variation in nematode community composition remains unexplained, likely reflecting the influence of unmeasured environmental factors or stochastic variation.

### Co-occurrence network analysis

**Fig 5:**
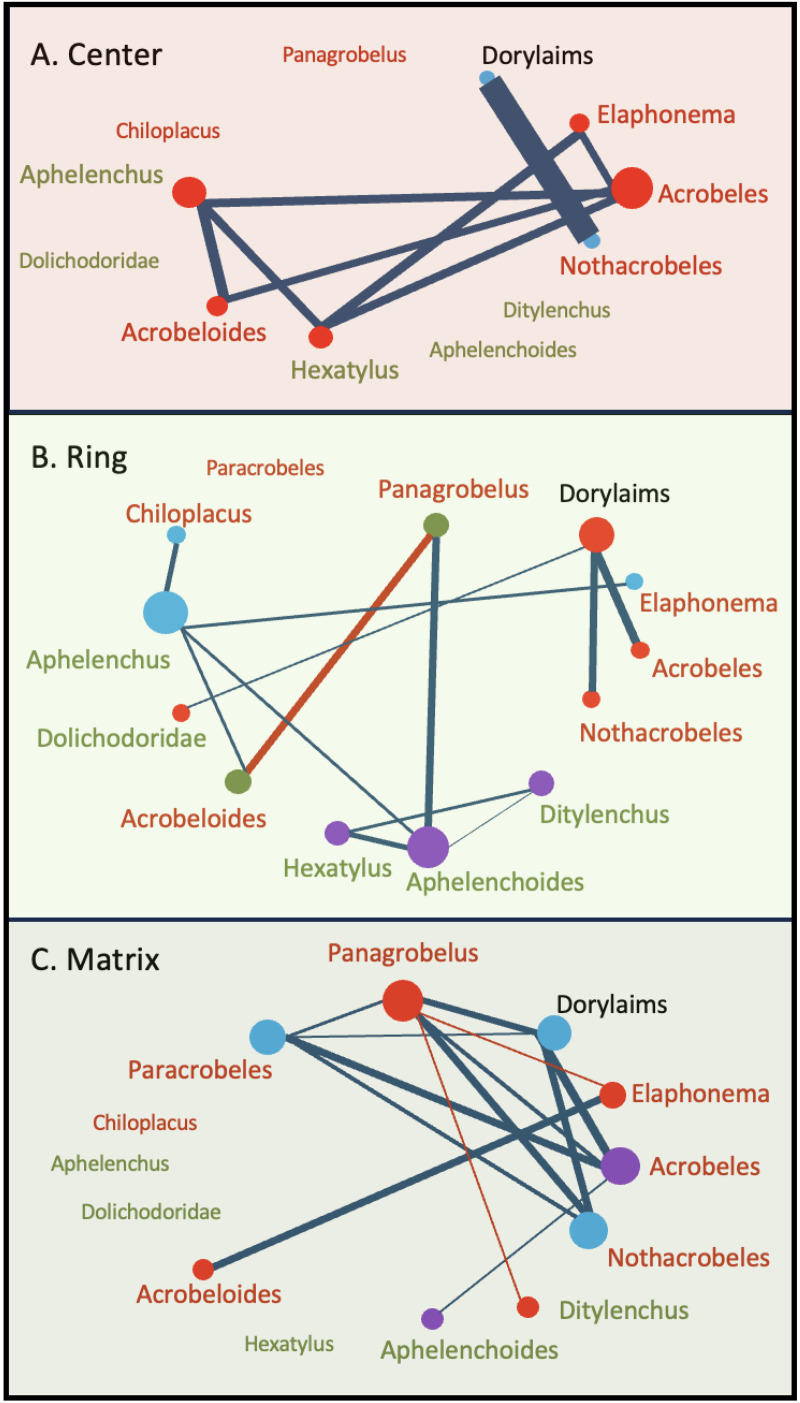
Network dynamics for nematode communities in fairy circle soils. Co-occurrence and correlation patterns were visualized using a threshold of 0.3 (positive correlations) and 0.2 (negative correlations) (all P < 0.05, S3 Table). Node size (circles) indicates the centrality of the taxon to the communities from each soil location, and node color indicates taxa that are most likely to share higher abundances, reflecting a common assemblage. Edges (lines) are weighted and colored according to the strength of these correlations, with negative correlations in red and positive in blue. The names of bacterial-feeding taxa are in red, and fungal and root-feeding taxa are in green (Dorylaims are omnivore/predators). Taxa shown without lines were present in the locations but did not show statistically meaningful correlations to any other taxa (S3 Table).

Network analysis showed that the abundances of many of the nematode taxa found were positively correlated with each other, suggesting that there is a core nematode community associated with fairy circle soils across the sampling sites and fairy circle positions (5A-C). Network analysis also revealed differences in how nematode communities assembled across the fairy circles. Center soils contained very few nematodes, but many of the taxa found there co-occurred, and their abundances were positively correlated with each other (*Aphelenchus*, *Acrobeloides*, *Hexatylus*, *Acrobeles*, and *Elaphonema*) (Fig 5A, S3 Table).

In ring soils, three different assemblages of nematodes emerged (Fig 5B). The fungal- and root hair-feeders *Aphelenchoides*, *Ditylenchus*, and *Hexatylus* co-occurred in many samples, with the abundances of each being positively correlated (Fig 5B, S3 Table). The stylet-bearing dorylaims and Dolichodoridae were commonly found with the bacterial-feeders *Nothacrobeles* and *Acrobeles* (Fig 5B). *Chiloplacus*, *Aphelenchus,* and *Elaphonema* also were a common assemblage (Fig 5B). In ring soils, there were no positive correlations among bacterial-feeding taxa (i.e., no edges, Fig 5B, S3 Table), and the abundance of *Panagrobelus* was negatively correlated to another bacterial-feeder, *Acrobeloides* (Fig 5B, S3 Table). Fungal-feeders showed more connectivity, with the abundance of *Aphelenchoides* positively correlated to *Aphelenchus* and *Hexatylus* (Fig 5B, S3 Table).

In matrix soils, Dorylaims, *Paracrobeles,* and *Nothacrobeles* formed one common assemblage (Fig 5C, S3 Table). *Acrobeles* and *Aphelenchoides* also co-occurred (Fig 5C). *Panagrobelus*, *Elaphonema*, *Acrobeloides*, and *Ditylenchus* also formed a common assemblage. Most bacterial-feeding taxa showed positive correlations in the matrix soils (Fig 5C, S3 Table). However, *Panagrobelus* was negatively correlated to *Elaphonema* (Fig 5C, S3 Table). Fungal-feeders showed no connections to each other in matrix soils (Fig 5C, S3 Table).

## Discussion

We hypothesized that nematode communities would differ among fairy circle positions due to their reliance on plant-derived resources, and our findings support this pattern. Nematode communities clearly reflected the distinct soil microhabitats created by the unique vegetation structure. Both abundance and diversity were higher in ring and matrix soils compared to the bare centers. Ring and matrix soils supported more fungal- and root-feeding taxa, while center soils were nearly defaunated and dominated by bacterial-feeding taxa. As expected, nematode abundance and diversity were positively correlated with soil organic matter content. Community composition also varied among the nine field sites and between microsites within fairy circles, with certain taxa responding to gradients in soil salinity (EC) and pH.

Ring and matrix soil nematode communities were similar to each other but more abundant and diverse than those found in the centers, demonstrating the connection nematodes have to vegetation. In arid systems, soils from under plants have often been shown to support greater soil nematode abundance and diversity than interplant soils due to the resource island effect on soil food webs [28,61,62]. In addition to supporting root feeding nematodes, plants enhance soil organic matter content, which is a primary determinant of the biomass and diversity of the bacterial and fungal communities that are consumed by microbivore nematodes [63]. Organic matter content has been shown to positively affect nematode abundance and diversity on global and local scales [28,64,65]. In fairy circles, soil organic matter content was highest under ring and matrix soils and positively correlated to nematode richness. This correlation was insignificant for the Simpson’s Diversity Index, suggesting that as organic matter increases, a few dominant taxa still make up most of the community. Interestingly, soil organic matter content was not found to explain a significant proportion of the variation in the distribution of nematode taxa in our RDA analysis. This may be because all nematodes respond similarly to higher organic matter or because variation in organic content across the samples was relatively limited at the time of sampling, potentially due to the drought conditions. Despite the lack of statistical connection to organic matter, some taxa of microbivorous nematodes were more abundant in ring and matrix soils, namely the bacterial-feeders *Panagrobleus* and *Acrobeles* and the fungal-feeders *Aphelenchus* and *Aphelenchoides*.

Soils from fairy circle rings and the matrix also contained more fungal and plant-parasitic nematodes. Network analysis identified a specific assemblage of fungal- and root-feeding nematodes (*Aphelenchoides*, *Ditylenchus*, and *Hexatylus*) that was common in ring soils (but not in center or matrix soils). The coexistence of these three taxa in many ring soils reflects their shared ability to exploit the enhanced grass growth and its effect on fungal biomass. While the density of nematodes in fairy circle soils was relatively low at the time of sampling, they have the potential to increase quickly during periods of plant growth in desert environments [28]. The low cp-values of the nematodes found in fairy circle soils suggest that the taxa present can rapidly respond to changing environmental conditions, such as the impact of a significant desert rainfall event that stimulates plant growth and nematode activity. Increasing numbers of root feeding nematodes could impact plant health and create water stress [38], contributing to a selective advantage for plants to self-organize into rings to reduce competition for water [15]. While we have found that nematode parasites are present in the ring soils, further experimentation is needed to determine the degree to which their activity could be contributing to the formation or persistence of fairy circles.

Interestingly, network analysis found no positive correlations with respect to abundance among bacterial-feeding nematodes in the ring soils. This suggests that there is some degree of niche partitioning occurring in this relatively resource-rich environment, perhaps due to environmental filtering or competition. In contrast, fungal-feeders were mainly positively correlated to each other in this same environment and appear to be able to co-exist in the ring soils, possibly by feeding on different fungi. In matrix soils, the opposite pattern was observed, with several bacterial-feeding taxa sharing correlated abundances but no fungal-feeders. Different assemblages of co-occurring nematodes were detected in the matrix as well, which hosts a greater plant diversity (if not biomass) during wet periods, which could alter resource quality and quantity. While the ring and matrix appear to share much in common as habitat for nematodes in this system, network analysis suggests they select for different nematode assemblages, which could impact soil food web function as it contributes to the formation and persistence of the *Stipagrostis* rings.

Soils from the bare fairy circle centers contained, on average, just 9 nematodes per 100 g of soil, while ring and matrix generally contained at least five times as many. Prior work has also found significantly lower microbial biomass in fairy circle centers [46]. These results are not likely due to the center soils being too dry or too hot compared to the ring and matrix. We measured very low soil moisture at all sites due to drought conditions (~ 0.3% moisture), and no differences were found between center, ring, or matrix soils (except at Tsiseb). However, prior work has shown that fairy circle centers can have higher soil moisture than in the rings, due to the lack of plant root uptake [2]. We did not measure soil temperature, but in a study at Namib Rand during the austral summer, the surfaces of center soils were found to be slightly cooler than the matrix (average daytime temperature of 45°C vs 47°C) [44]. Lower temperatures in the centers were attributed to higher moisture content generating more evaporative cooling than in the vegetated rings [44]. Overall, the low abundance and diversity of nematodes that we found in the center is correlated to the low organic matter content of those soils. The presence of wind ripples on the sand surface (Fig 1A) suggests ongoing disturbance is occurring, and center soils contain more coarse particles than ring soils due to wind-driven sediment transport [25,45]. Non-vegetated dune soils in the Namib Desert also contain very few nematodes, supporting the hypothesis that wind disturbance contributes to habitat unsuitability [61]. The presence and activity of microbivorous nematodes is important for plant productivity because these nematodes mineralize nutrients acquired by bacteria and fungi during decomposition, making them available for plant uptake [39]. The low abundance of microbes and nematodes in centers of fairy circles suggests that the soil food web is poorly functioning and unlikely to support plant growth, which may contribute to the persistent absence of vegetation.

The small number of nematodes that were found in centers could be part of established populations that feed and reproduce there, rather than transients that were blown there from ring or matrix soils. We found that *Acrobeloides*, a bacterial-feeder, is over-represented in the center soil communities compared to the vegetated soils. The centers of fairy circles have been shown to harbor distinct phylotypes of bacteria [24], and these may be preferred by *Acrobeloides*. Alternatively, some taxa of free-living soil nematodes show associations with insect bodies [66] and are known to engage in phoresy or to consume bacteria within corpses. Species of *Acrobeloides* have been found in association with insects in other ecosystems, including termites [67–70]. A possible interaction of this nematode with the termites or ants that are abundant in fairy circles warrants future investigation. Termites, which occur throughout the range of fairy circle distribution in Namibia, have been investigated as ecosystem engineers that prevent plant growth in fairy circle centers to create a water reservoir in deeper soil layers that they can then exploit [2,10]. We did observe both insects at the sites, although we did not quantify their presence.

Edaphic conditions can act as environmental filters, favoring distinct assemblages of nematodes depending on their tolerance ranges. In addition to being dry and low in organic matter, desert soils generally have high pH and salinities due to evaporation exceeding precipitation [71]. In this study, vegetated soils had higher electrical conductivity and lower pH than bare soils due to plant evapotranspiration and organic matter acidifying the soil. These changes in edaphic factors across the fairy circle pattern appear to play a role in shaping nematode community composition. According to RDA, site, position, pH, and EC explained 41% of the total variation in taxa data, a proportion consistent with findings from other ecological studies involving complex soil or benthic communities, where a large share of the variation is often driven by unmeasured biotic or abiotic factors [72]. The factors that support the co-existence of many nematodes within a trophic group are not well understood, but niche differentiation based on environmental gradients and resource availability is likely to play a role. In fairy circles, RDA suggested that some bacterial-feeders specialize for the microhabitats created by the vegetation pattern. *Panagrobelus and Acrobeles* were more abundant in soils with higher EC (mainly ring and matrix soils). *Acrobeloides* and *Elaphonema* were more abundant in higher pH soils (mainly center soils). Network analysis also demonstrated that *Panagrobelus* has a negative correlation with *Elaphonema* in matrix soils and *Acrobeloides* in ring soils. Biotic interactions, such as avoiding competition for a shared food source, and abiotic factors, such as preferences for specific ranges of edaphic factors, are likely contributing to these specializations. Habitat differentiation in bacterial-feeding nematodes has also been seen in the polar deserts of Antarctica, where the bacterial-feeder *Scottnema* was found primarily in the driest, lowest organic matter and high salinity soils, and *Plectus* occurs in soils with higher moisture [73,74]. Like *Plectus* and *Scottnema* in Antarctica, bacterial-feeders in the Namib Desert fairy circles seem to be showing habitat specialization based on biotic and abiotic factors.

This study is the first to investigate the biodiversity of soil fauna across a broad geographical range of Namibian fairy circles and the first to examine nematode communities associated with this unique vegetation pattern. However, the current study represents a snapshot of diversity in a specific layer of the soil during a single time period. We focused on the uppermost layer of soil because we wanted to study the environment where plants germinate and die, resulting in the bare centers of fairy circles [75], and where significant grass litter decomposition occurs [76–77]. Nematodes may be present in deeper layers of the soil, following the plant roots [78], although *Stipagrostis* grasses in the Namib Desert are generally shallow-rooted (0–20 cm) to take advantage of brief pulses of moisture [79]. We did conduct some deeper sampling at one site (10–20 cm, Farm Bloemhof), where we found similar abundances of nematodes to the upper layer in center soils, but with higher numbers in the deeper layers of ring soils (mainly more *Aphelenchus,* a fungal-feeder). Deeper soil sampling and/or direct sampling of roots for extraction of nematodes may elucidate a broader diversity of nematodes, including more plant parasitic taxa that can influence plant water stress. Furthermore, this study was conducted during an extended drought period, and study of the nematodes associated with fairy circles in a year when the grasses are actively growing is likely to provide a larger number of individuals, allowing for finer-scale identification and analyses. Finally, a study of the nematodes associated with arthropods at fairy circles may reveal a new layer of complexity to the ecological interactions underpinning fairy circles.

## Conclusions

Soil nematode communities show distinct configurations that are connected to fairy circles due to the impact of plants on soil organic matter, pH, and salinity. The presence of plant-parasitic taxa in fairy circle ring and matrix soils may contribute to plant water stress during growth periods and support the self-organization of *Stipagrostis* spp. to reduce competition for water. Soils from the bare fairy circle centers were nearly defaunated, suggesting that the same mechanism that limits establishment of plants within the circles is also affecting soil nematodes, either directly or indirectly. The near absence of nematodes in fairy circle centers indicates poor soil food web functioning that contributes to the hostile environment of the fairy circle centers for plants as well as nematodes. The mechanisms behind the creation and maintenance of fairy circles remain enigmatic, but this study demonstrates that these formations have belowground impacts that may contribute to sustaining the pattern.

## Supporting information

S1 Table Soil properties among fairy circle positions at nine field sites. (PDF)

S2 Table Distribution of nematode taxa among fairy circle field sites. (PDF)

S3 Table Pearson’s correlation test statistics for nematode taxa. (PDF)

S1 Fig Nematode abundance in fairy circle soils from nine field sites. Boxplots represent the interquartile range with lines representing the median value. Whiskers represent the minimum/maximum values, and circles are outliers. n = 6 samples per position. Lowercase letters indicate statistically significant differences among positions (N.S. = not significant, Kruskal-Wallis and Dunn’s Test, P* *< 0.05). a = center, b = ring, and c = matrix soils.
(JPG)

S2 Fig Simpson’s Index of diversity for nematode communities in fairy circle soils from nine field sites. Boxplots represent the interquartile range with lines representing the median value. Whiskers represent the minimum/maximum values, and circles are outliers. n = 6 samples per position. Lowercase letters indicate statistically significant differences among positions (N.S. = not significant, Kruskal-Wallis and Dunn’s Test, P* *< 0.05). a = center, b = ring, and c = matrix soils.
(JPG)
