# Supplementary material for: Namibian fairy circles: Hostile territory for soil nematodes
Source: PLoS One. 2025 Aug 12;20(8):e0315884. doi: 10.1371/journal.pone.0315884 (PMC12342241; doi:10.1371/journal.pone.0315884)
Supplement: S1 Table — (PDF) [file pone.0315884.s001.pdf]

S1 Table Soil properties by site:

**Organic matter content (%)***									
	M.fluss	Giribes	Twyfel.	Tsiseb	F.Bloem.	N.Nauk.	Rostock	Tsondab	N.Rand
Center	0.07±0.01b	0.20±0.04b	0.25±0.02b	0.28±0.05b	0.24±0.02b	0.33±0.02b	0.34±0.07a	0.21±0.02b	0.41±0.03a
Ring	0.23±0.03a	0.40±0.03a	0.35±0.02a	0.55±0.03a	0.32±0.01a	0.40±0.02ab	0.31±0.02a	0.34±0.05a	0.45±0.03a
Matrix	0.26±0.03a	0.33±0.03a	0.36±0.02a	0.35±0.03b	0.29±0.02ab	0.43±0.02a	0.31±0.03a	0.27±0.02ab	0.47±0.01a
P-value	0.0002	0.004	0.008	0.0004	0.017	0.024	0.84	0.029	0.22
**Electrical conductivity (µS/cm)***									
Center	71.33±3.3b	74.17±4.7b	116.00±15.4a	118.17±42.0a	101.50±8.4b	163.33±6.8b	127.33±6.1a	103.67±2.5a	77.50±4.1a
Ring	157.83±9.6a	185.33±17.7a	164.83±10.2a	191.83±28.7a	235.83±16.2a	185.00±11.8a	147.67±11.5a	113.83±4.4a	111.33±19.7a
Matrix	165.00±5.9a	193.17±19.9a	166.33±21.4a	100.17±6.1a	178.00±34.4a	180.33±4.9b	123.67±4.6a	107.50±5.1a	74.33±1.8a
P-value	<0.0001	<0.0001	0.034	0.052	0.0004	0.001	0.11	0.24	0.048
**Moisture (%)#**									
Center	0.21±0.02a	0.40±0.02a	0.33±0.04a	0.62±0.02a	0.26±0.03a	0.37±0.02a	0.32±0.03a	0.40±0.02a	0.10±0.01a
Ring	0.21±0.01a	0.32±0.01a	0.26±0.02a	0.48±0.02b	0.25±0.01a	0.38±0.02a	0.30±0.04a	0.45±0.02a	0.09±0.003a
Matrix	0.20±0.01a	0.32±0.03a	0.28±0.02a	0.55±0.04ab	0.23±0.02a	0.37±0.02a	0.33±0.02a	0.42±0.01a	0.11±0.004a
P-value	0.81	0.09	0.32	0.016	0.65	0.57	0.69	0.21	0.06
**pH#**									
Center	7.33±0.06a	8.03±0.10a	8.72±0.02a	8.25±0.17a	7.93±0.06a	8.88±0.04a	8.90±0.04a	8.80±0.12a	7.58±0.10a
Ring	7.30±0.07a	7.65±0.09b	8.42±0.05b	7.80±0.17a	7.92±0.06a	8.70±0.05a	8.78±0.07a	8.62±0.11a	7.57±0.07a
Matrix	7.37±0.10a	7.65±0.10b	8.48±0.07b	8.18±0.07a	7.87±0.10a	8.85±0.07a	8.82±0.04a	8.48±0.13a	7.67±0.07a
P-value	0.91	0.03	0.003	0.12	0.93	0.07	0.55	0.11	0.67

*Values represent the mean ± the standard error of the mean (n = 6). Within each site, lowercase letters indicate statistically significant differences among positions (one-way ANOVA, Tukey's HSD). #Values represent the mean ± the standard error of the mean (n = 6). Within each site, lowercase letters indicate statistically significant differences among positions (N.S. = not significant, Kruskal-Wallis and Dunn's Test).
