# Supplementary material for: Namibian fairy circles: Hostile territory for soil nematodes
Source: PLoS One. 2025 Aug 12;20(8):e0315884. doi: 10.1371/journal.pone.0315884 (PMC12342241; doi:10.1371/journal.pone.0315884)
Supplement: S2 Table — (PDF) [file pone.0315884.s002.pdf]

S2 Table Distribution of nematode taxa among fairy circle field sites*

			Marien.			Giribes			Twyfel.			Tsiseb			Farm Bloem.			NamibNauk.			Rostock			Tsondab			Namib Rand		
Taxon	Trophic Group#	cp-value	C	R	M	C	R	M	C	R	M	C	R	M	C	R	M	C	R	M	C	R	M	C	R	M	C	R	M
Acrobeles	BF	2	+	+	+		+	+	+	+	+	+	+	+	+	+	+	+	+	+		+	+	+	+	+	+	+	+
Acrobeloides	BF	2	+	+	+	+	+	+	+	+	+	+	+	+	+	+	+	+	+	+	+	+	+	+	+	+	+	+	+
Chiloplacus	BF	2		+	+						+		+	+	+			+	+	+		+			+	+		+	+
Drilocephalobus	BF	2					+	+			+		+	+				+											+
Elaphonema	BF	2	+	+	+	+	+	+	+	+	+	+	+	+				+	+	+		+	+				+		+
Mesorhabditis	BF	1								+														+	+				
Nothacrobeles	BF	2	+	+	+																								
Panagrobelus	BF	1	+	+	+		+	+							+	+	+					+		+	+	+		+	
Paracrobeles	BF	2		+	+		+	+														+			+				
Pelodera	BF	1	+													+													
Plectus	BF	2		+	+																								+
Aphelenchoides	FF	2		+	+		+	+	+	+	+		+	+	+	+	+	+	+	+	+	+			+	+		+	+
Aphelenchus	FF	2	+	+	+		+	+		+	+		+	+		+	+	+	+	+	+	+	+	+	+	+	+	+	+
Ditylenchus	FF/ PP	2					+	+						+					+	+	+	+		+	+	+	+	+	+
Hexatylus	FF/Insect parasite	2		+			+	+		+	+		+	+				+	+	+		+	+		+	+	+	+	+
Dolichodoridae	PP	3		+		+							+	+							+							+	+
Dorylaims	OM/P	4	+	+	+								+												+				
Discolaimus	P	4									+																		
Carcharolaimus	P	4									+														+				

*"+" indicates presence at the site, "C" = center of fairy circle, "R" = ring of circle, "M" = matrix surrounding circle) #BF = Bacterial-feeder, FF = Fungal-feeder, PP = Root feeder/Plant-parasitic, OM=omnivorous, P = Predator
