# Supplementary material for: Namibian fairy circles: Hostile territory for soil nematodes
Source: PLoS One. 2025 Aug 12;20(8):e0315884. doi: 10.1371/journal.pone.0315884 (PMC12342241; doi:10.1371/journal.pone.0315884)
Supplement: S3 Table — (PDF) [file pone.0315884.s003.pdf]

S3 Table Pearson's correlation test statistics (ρ and P) used for Network Analysis

Center soils:

ρ

	Panagrobelus	Acrobeles	Acrobeloides	Chiloplacus	Elaphonema	Nothacrobeles	Hexatylus	Aphelenchus	Aphelenchoides	Ditylenchus	Dolichodoridae	Dorylaims
Panagrobelus	1.00	-0.14	0.23	-0.09	0.04	-0.05	-0.09	0.15	-0.10	-0.04	-0.05	-0.05
Acrobeles	-0.14	1.00	0.26	0.28	0.27	-0.10	0.34	0.30	-0.09	0.14	-0.10	-0.10
Acrobeloides	0.23	0.26	1.00	0.10	0.10	0.12	0.18	0.44	0.06	0.16	-0.21	0.12
Chiloplacus	-0.09	0.28	0.10	1.00	0.07	-0.04	-0.07	0.09	0.23	0.03	-0.04	-0.04
Elaphonema	0.04	0.27	0.10	0.07	1.00	0.17	0.41	0.10	0.06	-0.06	0.12	0.17
Nothacrobeles	-0.05	-0.10	0.12	-0.04	0.17	1.00	-0.04	0.28	-0.04	-0.08	-0.02	1.00
Hexatylus	-0.09	0.34	0.18	-0.07	0.41	-0.04	1.00	0.32	-0.08	0.28	-0.04	-0.04
Aphelenchus	0.15	0.30	0.44	0.09	0.10	0.28	0.32	1.00	0.04	0.24	-0.07	0.28
Aphelenchoides	-0.10	-0.09	0.06	0.23	0.06	-0.04	-0.08	0.04	1.00	0.02	-0.04	-0.04
Ditylenchus	-0.04	0.14	0.16	0.03	-0.06	-0.08	0.28	0.24	0.02	1.00	-0.08	-0.08
Dolichodoridae	-0.05	-0.10	-0.21	-0.04	0.12	-0.02	-0.04	-0.07	-0.04	-0.08	1.00	-0.02
Dorylaims	-0.05	-0.10	0.12	-0.04	0.17	1.00	-0.04	0.28	-0.04	-0.08	-0.02	1.00

P

	Panagrobelus	Acrobeles	Acrobeloides	Chiloplacus	Elaphonema	Nothacrobeles	Hexatylus	Aphelenchus	Aphelenchoides	Ditylenchus	Dolichodoridae	Dorylaims
Panagrobelus		0.3516	0.1132	0.5570	0.7720	0.7402	0.5572	0.2985	0.4929	0.7927	0.7402	0.7402
Acrobeles	0.3516		0.0722	0.0539	0.0649	0.4838	0.0160	0.0366	0.5268	0.3216	0.4838	0.4838
Acrobeloides	0.1132	0.0722		0.4807	0.5135	0.3981	0.2265	0.0017	0.6626	0.2709	0.1458	0.3981
Chiloplacus	0.5570	0.0539	0.4807		0.6190	0.8015	0.6563	0.5202	0.1147	0.8221	0.8015	0.8015
Elaphonema	0.7720	0.0649	0.5135	0.6190		0.2531	0.0039	0.4930	0.6599	0.6860	0.4189	0.2531
Nothacrobeles	0.7402	0.4838	0.3981	0.8015	0.2531		0.8016	0.0490	0.7692	0.5996	0.8870	0.0000
Hexatylus	0.5572	0.0160	0.2265	0.6563	0.0039	0.8016		0.0230	0.6035	0.0492	0.8016	0.8016
Aphelenchus	0.2985	0.0366	0.0017	0.5202	0.4930	0.0490	0.0230		0.7801	0.1029	0.6205	0.0490
Aphelenchoides	0.4929	0.5268	0.6626	0.1147	0.6599	0.7692	0.6035	0.7801		0.8835	0.7692	0.7692
Ditylenchus	0.7927	0.3216	0.2709	0.8221	0.6860	0.5996	0.0492	0.1029	0.8835		0.5996	0.5996
Dolichodoridae	0.7402	0.4838	0.1458	0.8015	0.4189	0.8870	0.8016	0.6205	0.7692	0.5996		0.8870
Dorylaims	0.7402	0.4838	0.3981	0.8015	0.2531	0.0000	0.8016	0.0490	0.7692	0.5996	0.8870	

Ring soils:

ρ

	Panagrobelus	Acrobeles	Acrobeloides	Chiloplacus	Elaphonema	Paracrobeles	Nothacrobeles	Hexatylus	Aphelenchus	Aphelenchoides	Ditylenchus	Dolichodoridae	Dorylaims
Panagrobelus	1.00	0.16	-0.38	-0.01	-0.08	0.12	0.28	0.16	0.24	0.44	0.07	-0.06	0.26
Acrobeles	0.16	1.00	0.15	0.14	-0.15	0.29	0.29	0.08	0.05	0.09	0.04	0.17	0.35
Acrobeloides	-0.38	0.15	1.00	0.24	0.29	-0.03	-0.11	-0.28	0.31	-0.12	-0.05	-0.05	-0.16
Chiloplacus	-0.01	0.14	0.24	1.00	0.05	0.21	0.07	0.07	0.32	0.13	0.03	-0.19	0.10
Elaphonema	-0.08	-0.15	0.29	0.05	1.00	0.14	0.06	-0.24	0.30	-0.23	-0.08	-0.07	-0.20
Paracrobeles	0.12	0.29	-0.03	0.21	0.14	1.00	0.18	0.18	0.17	-0.11	0.08	0.07	0.10
Nothacrobeles	0.28	0.29	-0.11	0.07	0.06	0.18	1.00	-0.18	0.16	-0.03	-0.19	0.20	0.46
Hexatylus	0.16	0.08	-0.28	0.07	-0.24	0.18	-0.18	1.00	0.05	0.45	0.45	0.03	0.15
Aphelenchus	0.24	0.05	0.31	0.32	0.30	0.17	0.16	0.05	1.00	0.36	0.23	-0.12	0.02
Aphelenchoides	0.44	0.09	-0.12	0.13	-0.23	-0.11	-0.03	0.45	0.36	1.00	0.47	-0.04	0.12
Ditylenchus	0.07	0.04	-0.05	0.03	-0.08	0.08	-0.19	0.45	0.23	0.47	1.00	0.06	-0.11
Dolichodoridae	-0.06	0.17	-0.05	-0.19	-0.07	0.07	0.20	0.03	-0.12	-0.04	0.06	1.00	0.35
Dorylaims	0.26	0.35	-0.16	0.10	-0.20	0.10	0.46	0.15	0.02	0.12	-0.11	0.35	1.00

P

	Panagrobelus	Acrobeles	Acrobeloides	Chiloplacus	Elaphonema	Paracrobeles	Nothacrobeles	Hexatylus	Aphelenchus	Aphelenchoides	Ditylenchus	Dolichodoridae	Dorylaims
Panagrobelus		0.2574	0.0045	0.9367	0.5582	0.3734	0.0374	0.2385	0.0766	0.0008	0.6284	0.6910	0.0571
Acrobeles	0.2574		0.2799	0.3128	0.2734	0.0319	0.0312	0.5757	0.7346	0.4952	0.7569	0.2139	0.0106
Acrobeloides	0.0045	0.2799		0.0824	0.0319	0.8317	0.4345	0.0385	0.0224	0.3866	0.7161	0.7035	0.2618
Chiloplacus	0.9367	0.3128	0.0824		0.7035	0.1316	0.6401	0.6059	0.0181	0.3646	0.8229	0.1580	0.4636
Elaphonema	0.5582	0.2734	0.0319	0.7035		0.3157	0.6473	0.0823	0.0271	0.0986	0.5849	0.5907	0.1565
Paracrobeles	0.3734	0.0319	0.8317	0.1316	0.3157		0.1917	0.1996	0.2317	0.4238	0.5689	0.6062	0.4833
Nothacrobeles	0.0374	0.0312	0.4345	0.6401	0.6473	0.1917		0.1931	0.2443	0.8214	0.1765	0.1450	0.0005
Hexatylus	0.2385	0.5757	0.0385	0.6059	0.0823	0.1996	0.1931		0.7073	0.0006	0.0007	0.8316	0.2683
Aphelenchus	0.0766	0.7346	0.0224	0.0181	0.0271	0.2317	0.2443	0.7073		0.0067	0.0899	0.3721	0.8866
Aphelenchoides	0.0008	0.4952	0.3866	0.3646	0.0986	0.4238	0.8214	0.0006	0.0067		0.0003	0.7538	0.4064
Ditylenchus	0.6284	0.7569	0.7161	0.8229	0.5849	0.5689	0.1765	0.0007	0.0899	0.0003		0.6703	0.4372
Dolichodoridae	0.6910	0.2139	0.7035	0.1580	0.5907	0.6062	0.1450	0.8316	0.3721	0.7538	0.6703		0.0095
Dorylaims	0.0571	0.0106	0.2618	0.4636	0.1565	0.4833	0.0005	0.2683	0.8866	0.4064	0.4372	0.0095	

Matrix soils:

ρ

	Panagrobelus	Acrobeles	Acrobeloides	Chiloplacus	Elaphonema	Paracrobeles	Nothacrobeles	Hexatylus	Aphelenchus	Aphelenchoides	Ditylenchus	Dolichodoridae	Dorylaims
Panagrobelus	1.00	0.44	-0.30	0.14	-0.37	0.44	0.42	-0.18	0.10	0.29	-0.31	-0.11	0.65
Acrobeles	0.44	1.00	0.05	-0.04	-0.10	0.36	0.28	-0.19	-0.13	0.32	0.03	-0.01	0.45
Acrobeloides	-0.30	0.05	1.00	0.21	0.44	-0.07	-0.13	0.05	0.14	0.13	-0.11	0.24	-0.26
Chiloplacus	0.14	-0.04	0.21	1.00	0.20	0.03	0.02	0.13	0.20	-0.14	-0.25	0.23	0.03
Elaphonema	-0.37	-0.10	0.44	0.20	1.00	-0.06	0.01	-0.10	-0.14	0.07	0.07	0.21	-0.16
Paracrobeles	0.44	0.36	-0.07	0.03	-0.06	1.00	0.35	-0.11	0.15	0.26	-0.07	-0.07	0.45
Nothacrobeles	0.42	0.28	-0.13	0.02	0.01	0.35	1.00	-0.08	0.01	0.04	-0.16	-0.05	0.32
Hexatylus	-0.18	-0.19	0.05	0.13	-0.10	-0.11	-0.08	1.00	0.21	-0.20	-0.11	-0.09	-0.11
Aphelenchus	0.10	-0.13	0.14	0.20	-0.14	0.15	0.01	0.21	1.00	0.11	-0.18	-0.10	0.02
Aphelenchoides	0.29	0.32	0.13	-0.14	0.07	0.26	0.04	-0.20	0.11	1.00	-0.03	-0.07	0.29
Ditylenchus	-0.31	0.03	-0.11	-0.25	0.07	-0.07	-0.16	-0.11	-0.18	-0.03	1.00	0.26	-0.22
Dolichodoridae	-0.11	-0.01	0.24	0.23	0.21	-0.07	-0.05	-0.09	-0.10	-0.07	0.26	1.00	-0.07
Dorylaims	0.65	0.45	-0.26	0.03	-0.16	0.45	0.32	-0.11	0.02	0.29	-0.22	-0.07	1.00

P

	Panagrobelus	Acrobeles	Acrobeloides	Chiloplacus	Elaphonema	Paracrobeles	Nothacrobeles	Hexatylus	Aphelenchus	Aphelenchoides	Ditylenchus	Dolichodoridae	Dorylaims
Panagrobelus		0.0009	0.0277	0.2961	0.0053	0.0009	0.0017	0.1868	0.4870	0.0342	0.0236	0.4084	0.0000
Acrobeles	0.0009		0.7374	0.7672	0.4911	0.0074	0.0390	0.1660	0.3339	0.0191	0.8327	0.9589	0.0006
Acrobeloides	0.0277	0.7374		0.1315	0.0009	0.6229	0.3358	0.7220	0.3175	0.3393	0.4137	0.0790	0.0529
Chiloplacus	0.2961	0.7672	0.1315		0.1571	0.8329	0.8925	0.3623	0.1416	0.3192	0.0628	0.0990	0.8455
Elaphonema	0.0053	0.4911	0.0009	0.1571		0.6437	0.9197	0.4679	0.3245	0.6259	0.6201	0.1278	0.2612
Paracrobeles	0.0009	0.0074	0.6229	0.8329	0.6437		0.0102	0.4334	0.2930	0.0556	0.6039	0.6225	0.0006
Nothacrobeles	0.0017	0.0390	0.3358	0.8925	0.9197	0.0102		0.5873	0.9266	0.7638	0.2607	0.7328	0.0189
Hexatylus	0.1868	0.1660	0.7220	0.3623	0.4679	0.4334	0.5873		0.1291	0.1489	0.4413	0.5018	0.4332
Aphelenchus	0.4870	0.3339	0.3175	0.1416	0.3245	0.2930	0.9266	0.1291		0.4356	0.2014	0.4902	0.8691
Aphelenchoides	0.0342	0.0191	0.3393	0.3192	0.6259	0.0556	0.7638	0.1489	0.4356		0.8568	0.5959	0.0341
Ditylenchus	0.0236	0.8327	0.4137	0.0628	0.6201	0.6039	0.2607	0.4413	0.2014	0.8568		0.0604	0.1025
Dolichodoridae	0.4084	0.9589	0.0790	0.0990	0.1278	0.6225	0.7328	0.5018	0.4902	0.5959	0.0604		0.6224
Dorylaims	0.0000	0.0006	0.0529	0.8455	0.2612	0.0006	0.0189	0.4332	0.8691	0.0341	0.1025	0.6224	
