# Supplementary figures and images for: Namibian fairy circles: Hostile territory for soil nematodes
Source: PLoS One. 2025 Aug 12;20(8):e0315884. doi: 10.1371/journal.pone.0315884 (PMC12342241; doi:10.1371/journal.pone.0315884)

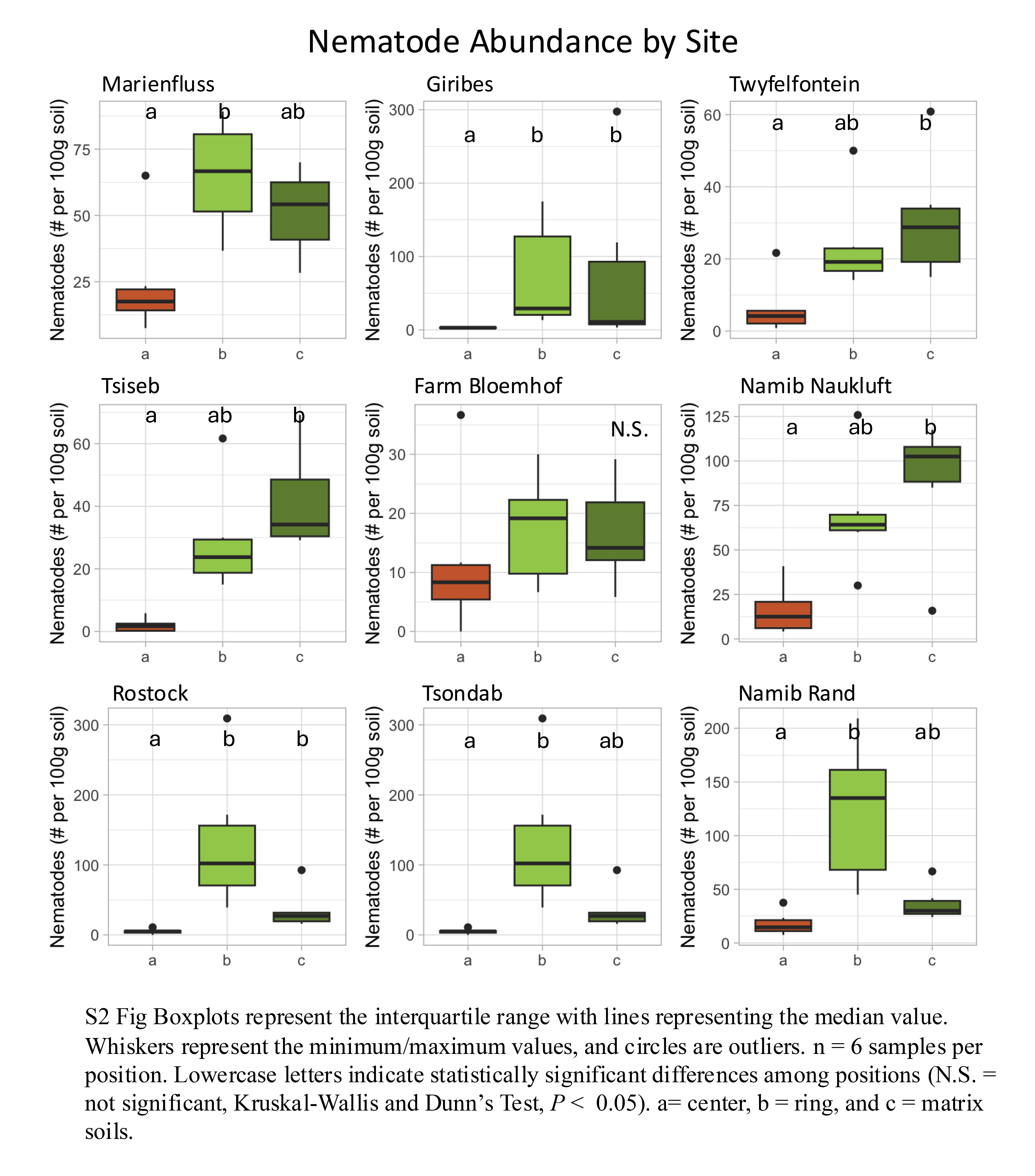

Supplement: S1 Fig — Boxplots represent the interquartile range with lines representing the median value. Whiskers represent the minimum/maximum values, and circles are outliers. n = 6 samples per position. Lowercase letters indicate statistically significant differences among positions (N.S. = not significant, Kruskal-Wallis and Dunn’s Test, P < 0.05). a = center, b = ring, and c = matrix soils. (JPG) [file pone.0315884.s004.jpg]

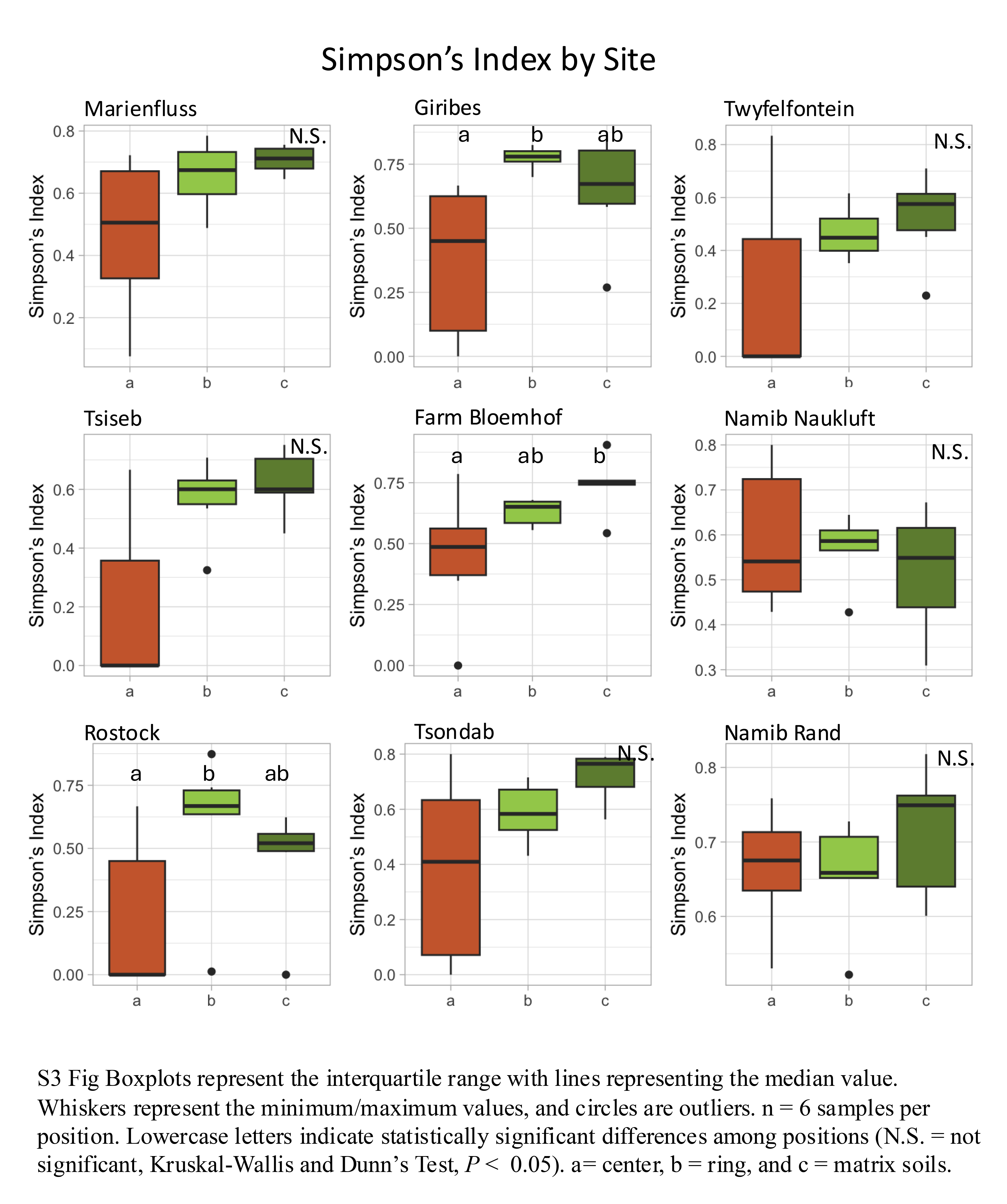

Supplement: S2 Fig — Boxplots represent the interquartile range with lines representing the median value. Whiskers represent the minimum/maximum values, and circles are outliers. n = 6 samples per position. Lowercase letters indicate statistically significant differences among positions (N.S. = not significant, Kruskal-Wallis and Dunn’s Test, P < 0.05). a = center, b = ring, and c = matrix soils. (JPG) [file pone.0315884.s005.jpg]
